# The Recently Enacted Law of New Jersey

**Published:** 1898-05

**Authors:** 


### THE RECENTLY ENACTED LAW OF NEW JERSEY.

Law in its relations to dentistry has been quite fully discussed upon the pages of this journal and elsewhere, but the subject is of such vital importance to the educational interests of dentistry that it cannot be set aside, for it is constantly recurring in new and startling phases, requiring constant watchfulness on the part of those whose duty it is to act as sentinels upon the outposts.

It has been assumed by the advocates of dental law that these efforts were made solely in the best interest of dental colleges and for the advance of dentistry, and many have been willing to accept this at the valuation placed upon it by the examining boards, State and national. Those more directly interested in educational work have been slow to recognize this doubtful interpretation, and have felt that the actuating impulse with many has been a desire to cripple the work of colleges, if not directly to antagonize their efforts. Whether this feeling has been justly founded or not is not material at the present writing, but the fact is quite prominent that if the boards are anxious to raise the dental profession to a higher standard, some of them, at least, have very peculiar methods in its manifestation.

The attention of the profession is called to the following clause in the recently enacted law of New Jersey, which reads as follows:

"No person shall be examined by the said Board unless he be twenty-one years of age, of good moral character, and having received a preliminary education *equal to that furnished by the common schools of the State* and be graduated in course with a dental degree from a dental school, college, or department of a university *recognized by said Board, or, unless he shall present the written recommendation of at least five licensed dentists of this State, of five years' standing, that he is qualified for such examination*, or shall hold a diploma or license conferring full right to practise dentistry in some foreign country and granted by some authority recognized by the Board." (Italics ours.)

When the National Association of Dental Faculties in 1884, immediately after the organization of that body, passed a law making it impossible for colleges under its jurisdiction to graduate a student upon what was termed "five years' previous practice," it was regarded everywhere as a marked advance. The rule of the colleges prior to this had become a scandal in the profession, and this action of the faculties was held to be in accordance with the wishes of the best elements in dentistry. The man whose entire experience may have been confined to five years in the dental laboratory, without the possibility of attaining any theoretical knowledge, was permitted under the five years' rule to matriculate, and after four months in the schools was sent out in the world as a Doctor of Dental Surgery. It was through a union of all the colleges that this stain upon American teaching was blotted out, it was hoped, forever. Gradually the standard of all the schools was raised, through the persistent efforts of the "faculties," to a course of three years, and it was then felt that the educational interests were placed upon a sure foundation.

The demand of the boards has been and still continues for a still higher standard, and some have gone so far that they have been obliged to retrace their steps.

The most persistent State in the Union to demand, through its Board, a higher standard has been New Jersey; indeed, this Board has recently declared it would not recognize any college that failed to come up to a standard far higher than that adopted by the National Association of Dental Faculties at its last meeting. In the face of this assertion, this same State has recently passed the law from which quotation has been made, which permits the Board to examine men upon the "recommendation of at least five licensed dentists," without a day of college training to their credit, and should they pass, are given the right to practise in the State of New Jersey. It may be said, by way of exculpation, that this is the law, and that the Board cannot do otherwise. Who made this law? The answer must be, the dentists of New Jersey. It is impossible for the governing dental power to shirk responsibility in this matter, even if so disposed, which is questionable.

It is deeply to be regretted that the dentists of New Jersey have had this scandalous piece of work thrust upon them, for we are willing to believe that all are not responsible. As it stands at present, New Jersey has put back dental education in that State to where it was prior to the organization of the National Association of Dental Faculties, and has undone within its borders the work of fourteen years.

If dentists needed an objective lesson upon the baleful effect of giving power to a few men, it has it in this clause of the law as quoted.

It is due to the dentists of the United States and the educational institutions that this obnoxious law be repealed as speedily as possible, and we have no doubt but that the best thinking men of New Jersey will take measures to effect this, for we are confident that there is a large body there who will not rest until this is accomplished.

